# Discrimination of candidate subgenome-specific loci by linkage map construction with an S_1_ population of octoploid strawberry (*Fragaria* × *ananassa*)

**DOI:** 10.1186/s12864-017-3762-y

**Published:** 2017-05-12

**Authors:** Soichiro Nagano, Kenta Shirasawa, Hideki Hirakawa, Fumi Maeda, Masami Ishikawa, Sachiko N. Isobe

**Affiliations:** 1 Kazusa DNA Research Institute, Kazusa-Kamatari 2-6-7, Kisarazu, Chiba 292-0818 Japan; 2 Chiba Prefectural Agriculture and Forestry Research Center, Chousei, Daizenno-Cyou 808, Midori, Chiba 299-4335 Japan; 3 Institute for Horticultural Plant Breeding, Kamishiki 2-5-1, Matsudo, Chiba 270-2221 Japan

**Keywords:** Cultivated strawberry, *Fragaria*, Genotyping array, Linkage map, Subgenome, Polyploidy, Single nucleotide polymorphism, Simple sequence repeat polymorphism

## Abstract

**Background:**

The strawberry, *Fragaria* × *ananassa*, is an allo-octoploid (2*n* = 8x = 56) and outcrossing species. Although it is the most widely consumed berry crop in the world, its complex genome structure has hindered its genetic and genomic analysis, and thus discrimination of subgenome-specific loci among the homoeologous chromosomes is needed. In the present study, we identified candidate subgenome-specific single nucleotide polymorphism (SNP) and simple sequence repeat (SSR) loci, and constructed a linkage map using an S_1_ mapping population of the cultivar ‘Reikou’ with an IStraw90 Axiom® SNP array and previously published SSR markers.

**Results:**

The ‘Reikou’ linkage map consisted of 11,574 loci (11,002 SNPs and 572 SSR loci) spanning 2816.5 cM of 31 linkage groups. The 11,574 loci were located on 4738 unique positions (bin) on the linkage map. Of the mapped loci, 8999 (8588 SNPs and 411 SSR loci) showed a 1:2:1 segregation ratio of AA:AB:BB allele, which suggested the possibility of deriving loci from candidate subgenome-specific sequences. In addition, 2575 loci (2414 SNPs and 161 SSR loci) showed a 3:1 segregation of AB:BB allele, indicating they were derived from homoeologous genomic sequences. Comparative analysis of the homoeologous linkage groups revealed differences in genome structure among the subgenomes.

**Conclusions:**

Our results suggest that candidate subgenome-specific loci are randomly located across the genomes, and that there are small- to large-scale structural variations among the subgenomes. The mapped SNPs and SSR loci on the linkage map are expected to be seed points for the construction of pseudomolecules in the octoploid strawberry.

**Electronic supplementary material:**

The online version of this article (doi:10.1186/s12864-017-3762-y) contains supplementary material, which is available to authorized users.

## Background

Polyploidy has long been recognized as one of the major forces in angiosperm evolution and diversification [1]. However, genetic and genomic analysis in polyploid species has fallen behind that in diploid species because of the complicated chromosomal composition and mode of inheritance in the former group. Polyploidy is generally classified into allo- and auto-polyploidy. Unlike those in auto-polyploid species, the homoeologous chromosomes in allo-polyploid species consist of subgenomes that make bivalent pairs by meiotic division. In this study, we classified loci identified by markers into subgenome specific and homoeologous loci; subgenome-specific locus has a specific target site of a marker existing only in one subgenome genomic regions, whereas homoeologous loci exist more than two subgenomes. Each subgenome includes subgenome-specific and homoeologous loci and the segregation patterns of loci in a cross population differ depending on the specificity of the chromosome. More specifically, subgenome-specific loci exhibit disomic inheritance, while the others show more complex segregation pattern. Therefore discrimination of the subgenome specificity of the locus is important for accurate genetic and genomic analysis.

The octoploid strawberry (*Fragaria* × *ananassa* Duchesne ex Rozier) is the most widely consumed berry crop in the world, and has a rich nutritional profile made up of minerals, vitamin C, folates and phenolic compounds [2]. *F.* × *ananassa* is commercially grown in temperate regions of the world and has an annual global production exceeding 10 million tons; in 2013 [3] 49.7% of the cultivated strawberries were produced in Asia, followed by North and South America (25.2%), Europe (19.2%), Africa (5.4%), and Oceania (0.5%). *F.* × *ananassa* is an allo-octoploid (2*n* = 8x = 56) and out-crossing species, with an estimated genome size of 1C = 708–720 Mb [4, 5] and 692 Mb [6]. Several models of the subgenome composition of the cultivated strawberry have been proposed. The AAA’A’ BBB’B’ model, which was proposed by Bringhurst [7], has been supported by molecular genetic studies (e.g., Sargent et al. [8] and the review by Kunihisa [9]) and was long recognized as a major genomic model of the cultivated strawberry. Recently, Sargent et al. [10] proposed the model A-A, b-b, X-X, X-X by construction of the SNP linkage map and discrimination of the ancestral chromosomes, where the A genome donor is an ancestral diploid *F. vesca*-like species, and the b and X subgenomes are from a hypothetical hexaploid derived from an *F. iinumae*-like and one or more unknown ancestral diploids, respectively. Though the discussion around the genome structure has continued, most researchers agree that the strawberry subgenome was derived from two or more ancestor species.

Genetic map construction have been proceeding towards whole genome decoding and molecular breeding in *F.* × *ananassa*. In the early stage, linkage maps of *F.* × *ananassa* were constructed with amplified fragment length polymorphism (AFLP), sequence tagged site (STS), random amplified polymorphic DNA (RAPD), and simple sequence repeat (SSR) markers [8, 11–15]. More recently, the whole genome sequences of *F. vesca* have become available [16], and the first high-throughput SNP genotyping platform for *F.* × *ananassa*, the IStraw90 Axiom® array, was developed by Bassil et al. [17]. Based on the Axiom® array, high density SNP linkage maps in the F_1_ populations were constructed with 6594 SNPs by Bassil et al. [17] or 8407 SNPs by Sargent et al. [10]. High density linkage maps were also constructed by double digest restriction-associated DNA sequencing (ddRAD seq; [18]) and diversity array technology (DArT; [19]). In most cases, these high density linkage maps and the early stage linkage maps were generated from F_1_ mapping populations, and the genome specificity within each homoeologous group (HG) was not discussed in detail.

The segregation patterns of polymorphic loci in mapping populations allow us to determine candidate genome-specific sequences in allo-polyploid genomes. That is, loci derived from subgenome-specific sequences show two homozygous genotypes (AA, BB) and the heterozygous genotype (AB), while those in the homoeologous sequences show either of the homozygous genotypes (AA or BB) and the heterozygous genotype. We considered that the detection of subgenome specificity would be easier in a self-inbred S_1_ population than an F_1_ population. This is because the genomes of an S_1_ population derived from two haploid genomes of single parental plant and subgenome-specific loci show AA:AB:BB = 1:2:1 segregation. Meanwhile genomes of an F_1_ population derived from four haploid genomes (each two from maternal and paternal parents), and the AA:AB:BB = 1:2:1 segregation was not theoretically observed. In the present study, we constructed a linkage map of an S_1_ mapping population with the IStraw90 Axiom® SNP array and SSR markers mapped onto the previously published integrated linkage map [15] for the identification of candidate subgenome-specific loci. The present linkage map was subsequently compared with the genome of *F. vesca* and previously constructed *F.* × *ananassa* SNP maps [10, 17] to survey the genome structure in *F.* × *ananassa*. The genetically closest linkage groups to the diploid *Fragaria* species, *F. vesca* and *F. iinumae*, were deduced based on the SNP haplotypes suggested by Sargent et al. [10]. The obtained result is expected to contribute to our understanding of the genome structure in *F.* × *ananassa*.

## Methods

### Plant materials and DNA extraction

The S_1_ population was developed by artificial self-pollination of an Japanese strawberry variety ‘Reikou’, which was bred at Chiba Prefectural Agriculture and Forestry Research Center in Japan. A flower bud was covered with a waterproof paper bag until receptacle maturation to prevent outcrossing. A total of 164 individuals in the S_1_ population were used for a linkage map construction. DNA was extracted from the young leaves with a DNeasy Plant Mini Kit (Qiagen Inc., Hilden, Germany), eluted into nuclease-free water and quantified with a spectrophotometer (NanoDrop ND1000; Nanodrop Technologies, DE, USA).

### SNP genotyping with the IStraw 90 Axiom® array

SNP genotyping of the S_1_ population was performed with the IStraw90 Axiom® SNP array (Affymetrix Inc., CA, USA) using the Affymetrix GeneTitan® system according to the manufacturer’s protocol. SNP calls were carried out using the Affymetrix Power Tools (APT) software. Called SNPs were classified into six categories by the APT software as follows: 1. Poly High Resolution (PHR): SNPs showing two homozygous (AA and BB) and a heterozygous genotype (AB) and passing all quality control steps (QC); 2. No Minor Homozygote (NMH): SNPs that passed all QC steps but had only two clusters, i.e., AA or BB and AB; 3. Off Target Variant (OTV): SNPs having an additional low intensity cluster resulting from slight mismatches between the probe and the sequences for that group of individuals; 4. Mono High Resolution (MHR): SNPs that passed all QC steps but were monomorphic; 5. CallRate Below Threshold (CRBT): SNPs in which the genotype call rate was under 97%; and 6. Other: SNPs for which the resultant SNP cluster pattern did not fall into any of the previous classes.

### Polymorphic analysis of SSR markers

A total of 1501 primer pairs of SSR markers mapped onto the previously published integrated SSR linkage map [15] were used for polymorphic analysis of the ‘Reikou’ S_1_ population (Additional file 1). PCR was performed in a 5 μl reaction volume using 0.6 ng of genomic DNA in 1× PCR buffer (Bioline, London, UK), 3 mM MgCl_2_, 0.08 U of BIOTAQ DNA polymerase (Bioline, London, UK), 0.8 mM dNTPs, and 0.4 mM of each primer. A modified touchdown PCR protocol was followed as described by Sato et al. [20]. The PCR products were separated by 10% polyacrylamide gel electrophoresis in tris-borate-ethylenediaminetetraacetic acid (TBE) buffer or with an ABI 3730xl fluorescent fragment analyzer (Applied Biosystems, MA, USA), according to the polymorphic fragment sizes of the PCR amplicons. The data were analyzed using the Polyans software package (http://www.kazusa.or.jp/phenotyping/polyans/) in the former case, and the GeneMarker software package (Softgenetics, PA, USA) in the latter case.

### Construction of the ‘Reikou’ linkage map

The PHR-SNPs that showed a segregation ratio of AA:AB:BB = 1:2:1 (*X* ^2^ ≥ 50 and missing data ≤ 20) were used for the initial grouping process and classified into seven groups according to the corresponding *F. vesca* ‘Hawaii 4’ v1.1 reference genome [21, 22]. The PHR-SNPs in each group were subsequently classified into multiple LGs using the color map method [23], which employed a comparison of graphical genotypes of the segregation data. During the process of color mapping, reciprocal genotypes were converted to coupling genotypes. The robustness of the data sets for each LG was then confirmed using the Grouping Module of the MultiPoint 3.3 (MultiQTL Ltd, Haifa, Israel) with a logarithm of odds (LOD) threshold of 2.0. Then, SNPs in each linkage group were ordered with the following parameters: a population type of F_2_, minimum LOD scores of 10.0, and maximum threshold of 0.25. After the first ordering, solitary missing data were imputed according to the genotypes of flanking SNPs. The residual PHR-SNPs, NMH-SNPs and polymorphic SSR loci were then added into each LG by the color mapping method. The robustness of each re-formed LG was confirmed again using the Grouping Module of the JoinMap program, version 4.0, with an LOD threshold of 2.0 (Kyazma, Wageningen, The Netherlands). The ordering was then performed with the following parameters: Haldane’s mapping function, LOD > 1.0, recombination frequency < 0.4, goodness of fit jump threshold for removal of loci = 5.0, number of added loci after which a ripple is performed = 1, and third round = yes. After the second ordering, loci showing dominant inheritance were added to the same genetic positions of mapped loci that showed the nearest orthologous positions on *F. vesca* (v2.0.a1) pseudomolecules.

### Comparison of the ‘Reikou’ linkage map with the *F. vesca* genome, an *F. iinumae* linkage map, and *F.* × *ananassa* SNP linkage maps

For comparison of the locus positions of the ‘Reikou’ linkage map with the *F. vesca* genome, a BLAST search was performed for the probe sequences of the SNPs of the IStraw90 Axiom® SNP array and the primer sequences of the SSR markers against *F. vesca* pseudomolecules version 2.0.a1 [24]. The corresponding SNPs and SSR locus positions showing top hit with a cut off E-value ≤ 1e-10 between the ‘Reikou’ linkage map and the *F. vesca* genome, and the corresponding SNPs between the ‘Reikou’ linkage map and the *F. iinumae* linkage map [25] were graphed using the program Circos [26]. The relationships between the physical positions on *Fvb* v2.0 pseudomolecules and the linkage positions of the SNPs and SSR locus on the ‘Reikou’ linkage map was generated by scatterplots with R software, version 3.2.3 [27]. The commonality of the mapped SNPs were investigated for the ‘Holiday’ × ‘Korona’ (HK) map [17] and the ‘Darselect’ × ‘Monterey’ (DM) map [10].

### Mapping Illumina genome reads

In order to verify the duplication of the SNPs in the genome, a total of 108.6 M Illumina reads, each of which was 101 bases in length, were mapped onto the probe sequences of the PHR and NMH-SNPs located on the ‘Reikou’ linkage map. The NGS reads from homoeologous sequences are mapped more frequent than that from subgenome specific sequences because homoeologous sequences have more occasions to be obtained by a massive parallel sequencing platform. The mapped reads were derived from the ‘Reikou’ genome and generated by Illumina GAIIx in the previous study [6]; data are available from the DDBJ Sequence Read Archive (DRA) under the accession number DRA001114. Read mapping was performed by using Bowtie2 software [28] with the local mode and very-sensitive options. The number of the mapped reads was counted for each probe sequence and scatterplots were generated by R software, version 3.2.3 [27]. The ratio of the probes for the mapped read depth onto the probe sequences was calculated for PHR- and NMH-SNPs.

### Comparison between the haploSNPs

The genetically closest linkage groups to *F. vesca* and *F. iinumae* were deduced based on the SNP haplotypes, which have been determined by comparison for SNP site among accessions and achieved a technical reduction in ploidy [17]. Here a SNP-SNP has been defined as a SNP with the destabilizing SNP site within 6 bp from the marker SNP site [17]. HaploSNP categories for each mapped SNP-SNP have been obtained by Sargent et al. [10] in comparisons based on whether the critical allele at the destabilization site matched or did not match the allele at the respective site in each of the two diploids, *F. vesca* and *F. iinumae*. Matched haploSNPs were counted up for four categories, Y-N (*F. vesca* matched but not *F. iinumae*), Y-Y (both *F. vesca* and *F. iinumae* matched), N-Y (*F. vesca* not matched but *F. iinumae* matched), and N-N (neither *F. vesca* nor *F. iinumae* matched), in each LG.

## Results

### Segregation pattern of polymorphic loci in *F.* × *ananassa*

**Fig. 1:**
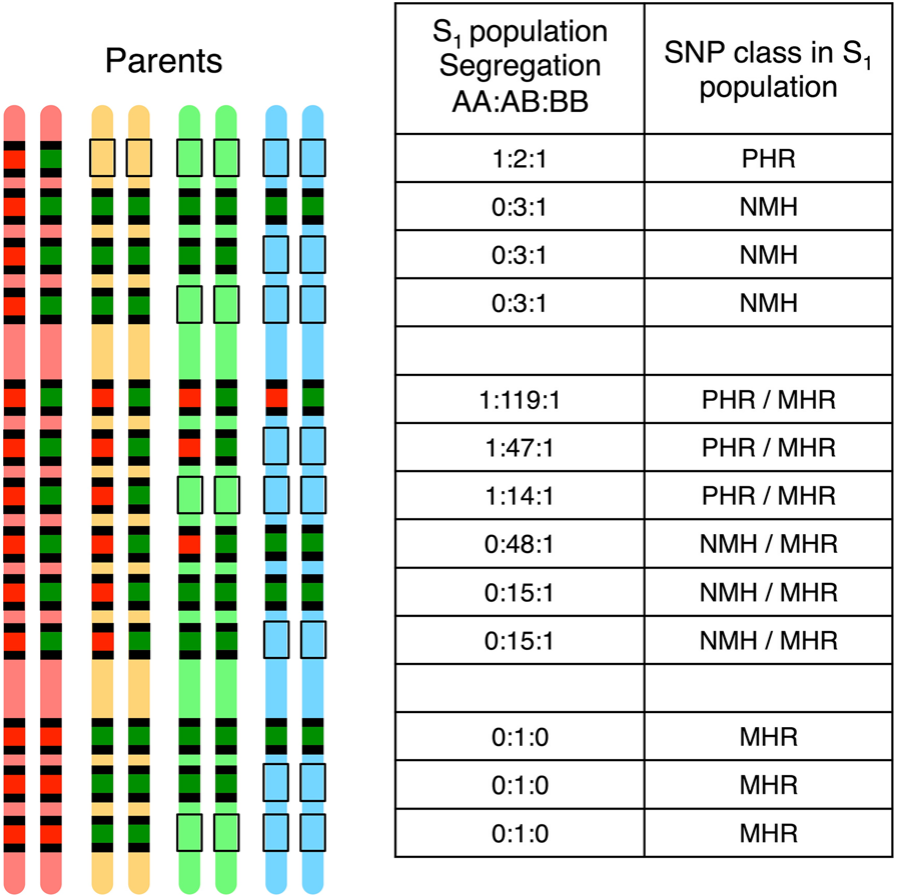
Expected segregation pattern of diallels in an S_1_ population of allo-octoploid species. *Pink*, *yellow*, *light green* and *aqua bars* represent the four subgenomes. *Red* and *green boxes* show alleles A and B, respectively. *Boxes* with *black lines* indicate the null alleles

The expected segregation patterns of polymorphic loci in S_1_ and F_1_ mapping populations in allo-octoploid species are shown in Fig. 1 and Additional file 2. The polymorphic locus derived from subgenome-specific sequences of an S_1_ population (ABOOOOOO) exhibits disomic segregation patterns of AA:AB:BB = 1:2:1. In contrast, the polymorphic loci in F_1_ population (ex. AAOOOOOO × ABOOOOOO) shows a 1:1 segregation pattern, which is the same as the segregation pattern of the polymorphic locus derived from non-subgenome-specific sequences (ex. AAAAOOOO × ABAAOOOO). AA:AB:BB = 1:2:1 segregations are observed in ABOOOOOO × ABOOOOOO cases in an F_1_ population, but the accuracy of mapped positions is generally lower because the phases of the AB genotypes are indeterminable. These facts indicate that the subgenome specificity is capable of distinguishing polymorphic loci by segregation pattern in an S_1_ population, but not always in an F_1_ population. The polymorphic loci derived from homoeologous sequences of an S_1_ population (ABBBOOOO, ABBBBBOO and ABBBBBBB) exhibit segregation patterns of AA:AB:BB = 0:3:1.

### Construction of the ‘Reikou’ linkage map

The 95,062 SNPs on the IStraw90 Axiom® array were classified into six categories according to their polymorphic or non-polymorphic behavior in the S_1_ population, i.e., 12,678 (13.3%) PHR (codominant segregation), 22,856 (24.0%) NMH (dominant segregation), 51,868 (54.6%) MHR (AA or BB), 449 (0.5%) OTV, 2817 (3.0%) CRBT and 4394 (4.6%) others (Table 1). Meanwhile, a total of 863 out of 1501 SSR markers showed polymorphisms in the S_1_ population (Additional file 1). Along with the 863 polymorphic SSR loci, the 12,678 PHR-SNPs and 22,856 NMH-SNPs were subjected to subsequent linkage analysis.

**Table 1:** Numbers of SNPs on the IStraw90 Axiom® array classified into the six categories

Classes	Number of SNPs	Ratio [%]	Number of mapped SNPs	Mapped ratio [%]
Poly High Resolution (PHR)	12,678	13.3	8,588	67.7
No Minor Homozygote (NMH)	22,856	24.0	2,414	10.6
Mono High Resolution (MHR)	51,868	54.6	-	-
Off Target Variant (OTV)	449	0.5	-	-
Call Rate Below Threshold (CRBT)	2,817	3.0	-	-
Other	4,394	4.6	-	-
Total	95,062	100.0	11,002	11.6

Number of SNPs, ratio of each category, number of mapped markers and mapped rates are presented. See also the texts about details of the six categories

A total of 11,574 loci, including 8588 PHR-SNPs, 2414 NMH-SNPs and 572 SSR loci, were mapped onto 31 LGs (Table 2, Additional file 3 and Additional file 4). The 11,574 loci were located on 4738 unique positions (bin) on the linkage map. The 572 SSR loci were derived from 519 SSR markers (Additional file 1), and classified into 411 codominant and 161 dominant SSR loci. As a result, the numbers of mapped co-dominant and dominant loci (including both SNPs and SSRs) were 8999 (77.8%) and 2575 (22.2%), respectively. Most of the mapped SNPs (10,181, 92.5%) were classified into the SNP-SNP category, which is one of the haplotype categories defined by Bassil et al. [17], and formed haplotypes with neighbor SNPs located within six bases from the target SNPs in probes.

**Table 2:** Summary statistics of the’Reikou’ linkage map

HG	Number of LGs	Total length [cM]	Mean length [cM]	Number of mapped loci									Mean bin span [cM]	*F. vesca* genome coverage [%] ^a)^
				Total	PHR-SNPs	[%]	NMH-SNPs	[%]	Codominant-SSR	[%]	Dominant-SSR	[%]		
1	5	394.0	78.8	1,111	815	73.4	235	21.2	49	4.4	12	1.1	0.94	75.2
2	4	423.4	105.9	1,628	1,178	72.4	347	21.3	73	4.5	30	1.8	0.80	75.5
3	4	446.1	111.5	1,824	1,357	74.4	383	21.0	64	3.5	20	1.1	0.77	82.9
4	4	320.0	80.0	996	718	72.1	211	21.2	40	4.0	27	2.7	1.26	64.6
5	4	382.2	95.5	1,793	1,314	73.3	411	22.9	48	2.7	20	1.1	0.56	76.8
6	6	481.0	80.2	2,377	1,801	75.8	474	19.9	69	2.9	33	1.4	0.51	91.6
7	4	369.9	92.5	1,845	1,405	76.2	353	19.1	68	3.7	19	1.0	0.53	95.3
Total	31	2,816.5	92.0	11,574	8,588	74.2	2,414	20.9	411	3.6	161	1.4	0.59	80.4

^a)^ Coverage ratio [%] of *F. vesca* pseudomolecules (Fvb v2.0.a1) by the ‘Reikou’ linkage map

Number of LGs, total linkage distances, mean linkage distance, numbers of SNP and SSR loci, ratio of PHR- and NMH-SNPs, ratio of codominant- and dominant-SSR loci, mean bin span, coverage for physical distances associated with each LGs on *F. vesca* (Fvb) v2.0 pseudomolecules are shown

The 31 LGs were classified into seven HGs according to the chromosomes of *F. vesca* (‘Hawaii 4’ v1.1 reference genome) where SNP probes were designed. Of the seven HGs, five (HG2, 3, 4, 5 and 7) consisted of four LGs, while HG1 and HG6 consisted of five and six LGs, respectively. Each LG was named using the corresponding chromosome number of *F. vesca*, along with letters that were assigned as follows. The LGs showing the highest similarity to *F. vesca* in each HG according to haploSNPs (as described in a later section) were assigned the letter A, and the letters B_I_ to B_IV_ were assigned according to the number of mapped loci, with B_I_ having the most and B_IV_ the fewest mapped loci. The two LGs in HG6 were named LG6A-1 and LG6A-2, because both showed highly similarity to *F. vesca*, as will be described in a later section. The length of each LG ranged from 51.2 cM (LG6B_III_) to 187.5 cM (LG2A), representing a total length of 2816.5 cM. The average distance between two mapped positions (bin) was 0.59 cM (Table 2 and Additional file 4). The mean segregation distortion ratio was 14.9%, ranging from 0% (LG1B_IV_) to 84.5% (LG3B_III_). The ratios of mapped PHR loci to total loci ranged from 67.8% (1B_IV_) to 90.8 (6A-1) with a mean value of 77.8%.

### Comparison with the *F. vesca* genome

**Fig. 2:**
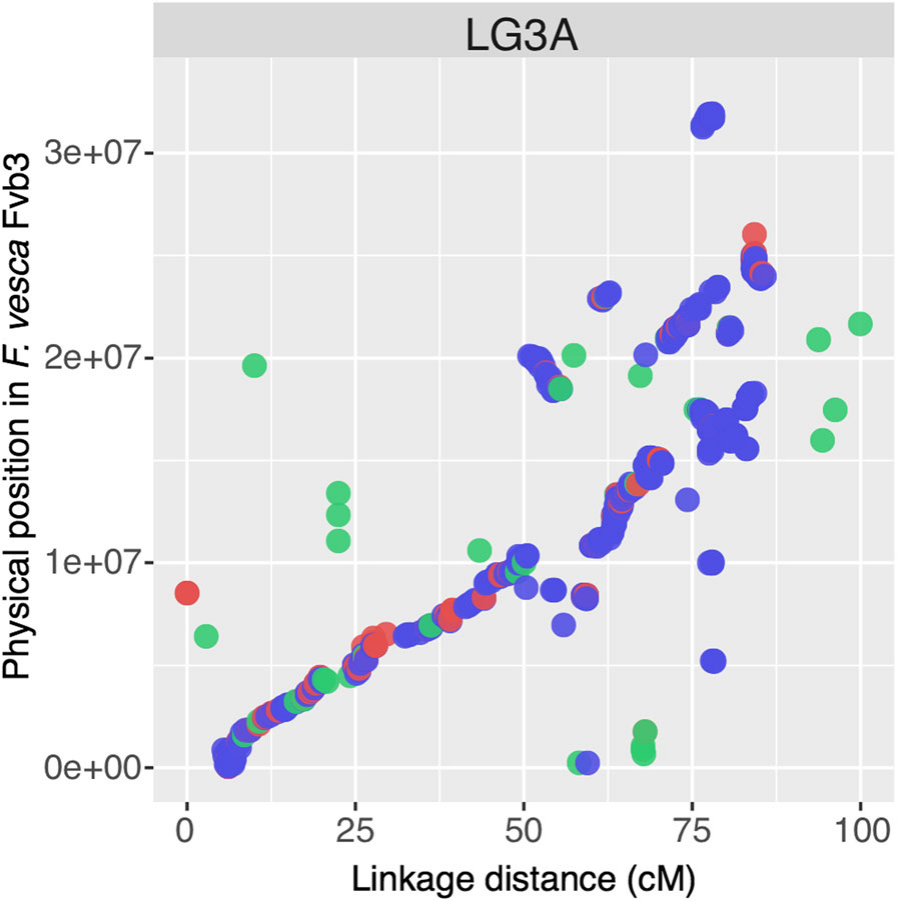
Comparison between the physical positions on *F. vesca* pseudomolecules (v2.0.a1) and the linkage positions of SNP and SSR loci on LG3A of the ‘Reikou’ linkage map. The PHR-SNP, NMH-SNP and SSR loci are shown with *blue*, *red* and *green dots*, respectively

Obvious collinearity was observed between the ‘Reikou’ linkage map and the *F. vesca* genome, but not between the ‘Reikou’ and *F. iinumae* linkage maps because of the limited number (51) of the commonly mapped SNPs (Additional file 5). The mean coverage ratio of LGs to the *F. vesca* genome (pseudomolecules) was 80.4%, with a range from 13.3% (LG1B_IV_) to 97.3% (LG3B_I_, Additional file 4). Local inversion and deletion were observed in many of the LGs (Fig. 2, Additional file 6). LGs belonging to HG5, 6, and 7 tended to show higher collinearity than LGs belonging to HG1, 2 and 3. Less collinearity was observed at the distal ends of LG1A and LG2A and the proximal end of LG2B_I_, and throughout the whole length of LG1B_IV_.

### Positions of candidate subgenome-specific SNPs

The PHR- and NMH-SNPs, representing candidate subgenome-specific and non-specific sequences, respectively, were randomly mapped across the LGs (Additional files 6 and 7). No clear distinction was observed between the mapped location of PHR- and NMH-SNPs. A total of 655,006 (0.60%) of the 108.6 M ‘Reikou’ Illumina reads were mapped onto the probe sequences of most (77.5%) of the mapped SNPs (Additional file 7). The average number of mapped reads across LGs was significantly higher in NMH-SNPs (68.9, *p* < 0.001, Student’s *t*-test) than in PHR-SNPs (56.9). In each LG, significant differences (*p* < 0.05, Student’s *t*-test) in mapped reads between PHR- and NMH-SNPs were identified in ten (Additional file 7, 1A, 1B_III_, 2A, 2B_I_, 3A, 5A, 6A, 6B_II_ 7A, and 7B_III_) of the 31 LGs. The ratio of the read depth for the mapped reads on the probe sequences of PHR-SNP showed a single peak, while that of NMH-SNP showed more than six peaks (Additional file 8).

**Fig. 3:**
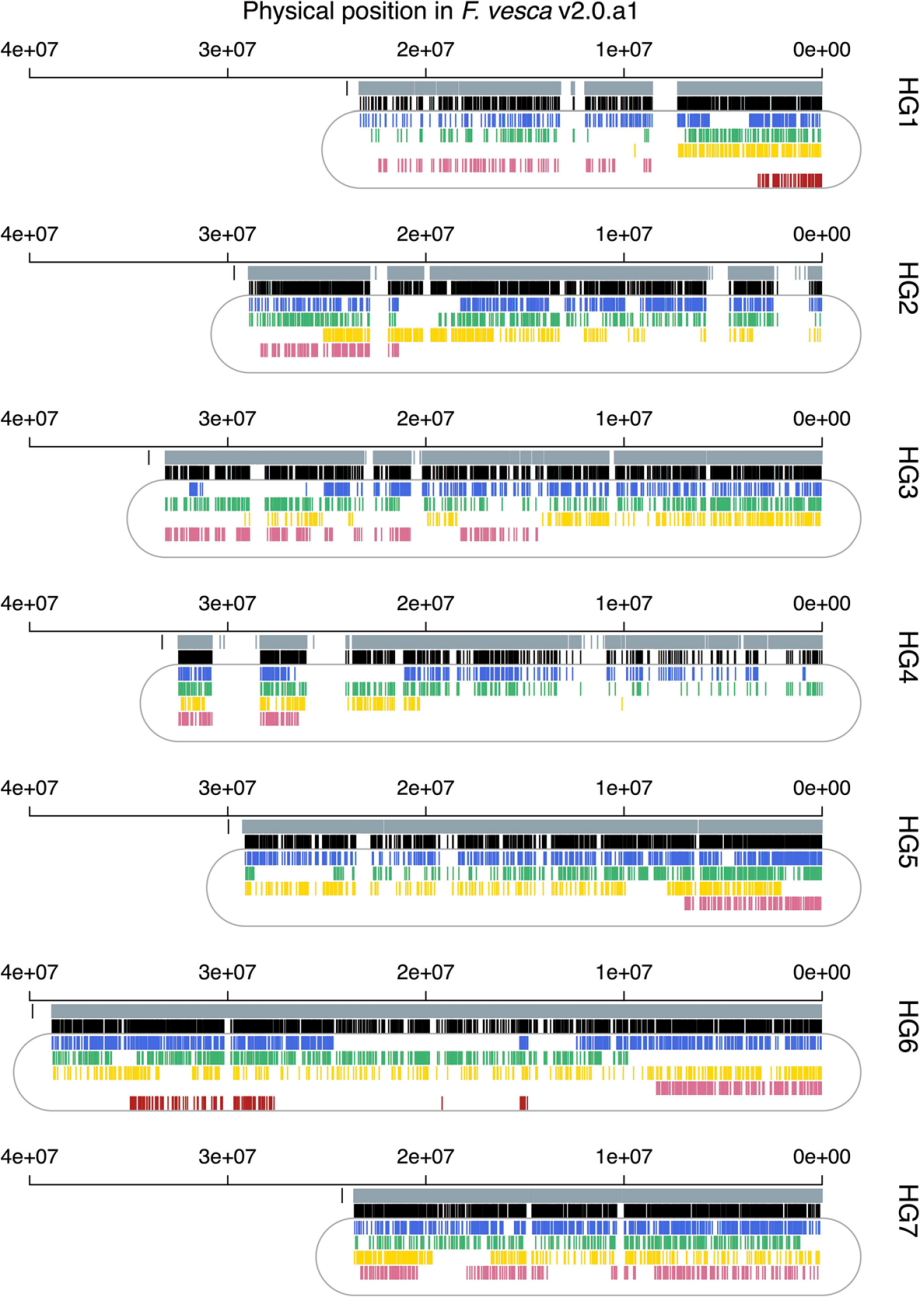
Physical positions on *F. vesca* pseudomolecules (v2.0.a1) of the designed and mapped PHR-SNPs on the ‘Reikou’ linkage map. All the SNPs designed on the Axiom® array and mapped PHR and NMH SNPs on HGs are shown with *gray* and *black bars*, respectively. Fragmentized LGs in HG6, LG6A-1 and LG6A-2 were jointed, because both of the LGs showed large numbers of Y-N haploSNPs corresponding to the *F. vesca* genome and mapped non-overlapped positions on the *F. vesc*a genome (see also the text). The *black line* at the distal end of the SNP designed position (*gray column*) shows the maximum length of the *F. vesca* genome v2.0.a1. The SNP positions of LG A, B_I_, B_II_, B_III_, and B_IV_ are shown by *blue*, *green*, *yellow*, *pink*, and *red* colored lines, respectively

The PHR-SNPs on the ‘Reikou’ linkage map showed their correspondence positions across the whole region of the *F. vesca* genome, and the distribution patterns were different among the LGs (Fig. 3). PHR-SNPs on all LGs in HG7, three (A, B_I_, and B_II_) LGs in HG2, HG3, HG5 and HG6, and two (A and B_I_) LGs in HG1 and HG5 were distributed throughout most of the region of the *F. vesca* chromosomes. Other LGs were located in distal regions of the *F. vesca* genome.

### Discrimination of the ancestral subgenome

Of the 11,002 mapped SNPs, 1183 were commonly mapped on the ‘Reikou’ linkage map and ‘Darselect’ × ‘Monterey’ linkage map [10] (DM, as described for the detail in a later section) and 1076 SNP-SNP were further classified into four haploSNPs according to the results of Sargent et al. [10]: Y-N (*F. vesca* matched but not *F. iinumae*), Y-Y (both *F. vesca* and *F. iinumae* matched), N-Y (*F. vesca* not matched but *F. iinumae* matched), and N-N (neither *F. vesca* nor *F. iinumae* matched). The Y-N haploSNPs, which corresponded to the *F. vesca* genome, were frequently observed on each single LG (LGA) in all HGs except HG6, and two shorter LGs (LG6A-1 and LG6A-2) in HG6 (Additional file 9). Since LG6A-1 and LG6A-2 collinearly showed similarity against the upper and lower regions of *Fvb*6 in *F. vesca* pseudomolecules v2.0.a1, we considered that these LGs could be connected and illustrated as a single LG (Fig. 3, Additional files 6 and 7). On the other hand, no LGs were specified that located a significantly large number of N-Y haploSNPs, which is corresponding to the *F. iinumae* genome, although N-N haploSNPs were frequency observed in multiple LGs of each HGs.

### Commonly mapped SNPs across the three mapping populations

The numbers of commonly mapped SNPs on the IStraw 90 Axiom® array were investigated among the ‘Reikou’ linkage map and the two previously published maps (‘Holiday’ × ‘Korona’ (HK), [17] and ‘Darselect’ × ‘Monterey’ (DM), [10], Additional file 10). A total of 22,207 SNPs were mapped onto each of the three linkage maps. Among this total, 416 SNPs were included on all three linkage maps; together these accounted for only 1.9% of the total mapped SNPs. In addition, 901 (4.1%), 1295 (5.8%), and 767 (3.5%) SNPs were commonly mapped between two of the three linkage maps of HK and DM, HK and ‘Reikou’ S_1_, and DM and ‘Reikou’ S_1_, respectively. Most of the mapped SNPs were population-specific: the numbers of specific SNPs in HK, DM, and the ‘Reikou’ S_1_ were 3981, 6323, and 8524, respectively.

## Discussion

### Discrimination of candidate subgenome-specific loci

In this study, we used an S_1_ population for linkage map construction to identify candidate subgenome-specific loci. In theory, polymorphic loci derived from subgenome-specific loci in an S_1_ population are expected to segregate into two homozygous genotypes and a heterozygous genotype and thus to be classified as PHR, while those derived from non-subgenome-specific loci are expected to show neither of the two homozygous genotypes and thus to be categorized as NMH loci [17]. In contrast, the Axiom® array system calls SNP genotypes according to the proportions of allele-specific signals. The IStraw90 Axiom® SNP array is designed to fit polyploid species, and sometimes can distinguish both homozygous genotypes (AA and BB) even if the SNPs are derived from non-subgenome-specific loci. In other words, there was a possibility that SNPs derived from non-subgenome-specific loci were included in PHR-SNPs, although the distribution for the coverage of the mapped reads (Additional file 8) suggests that most of the SNPs derived from subgenome-specific loci were included in PHR-SNPs. Therefore, we considered that the PHR loci on the present map represented “candidate” subgenome-specific loci.

The candidate subgenome-specific loci marked by PHR loci were distributed throughout the linkage map of ‘Reikou’, and no clear distinction was observed between these loci and the non-subgenome-specific loci. Meanwhile, orthologous regions between the LGs and the *F. vesca* genome were observed different positions among the LGs in the same HGs. In particular, SNPs mapped onto the LGs designated with the letter B_III_ (or sometimes B_II_ or B_IV_) showed similarity against limited loci of the *F. vesca* genome. This suggested that there were large differences among the genomes, though there is a possibility that several regions of the ‘Reikou’ genomes were missing from the linkage map due to the monomorphic structure among the four subgenomes. In addition, re-arrangements in various chromosomes were observed between each LG and the *F. vesca* genome. Meanwhile, it should be mentioned that some disagreements with the *F. vesca* genome could be due to errors in, or the incompleteness of, the Hawaii 4 genome assemblies and not to actual rearrangements. Based on the results obtained in the present study, we concluded that subgenome-specific sequences were randomly located across the *F. × ananassa* genome, and in parallel, small-to-large scale differences existed among the four subgenomes.

In the Axiom® array, 19 octoploid strawberry accessions (15 *F.* × *ananassa* lines and one and three accessions from the ancestral octoploids, *F. virginiana* and *F. chiloensis,* respectively) and one diploid *F. iinumae* accession were used for variant discovery with reference of the *F. vesca* Hawaii 4 v1.1 genome [17]). Large differences in LG length were also observed in the previous linkage maps [10, 17] but not in the genome-wide DArT marker linkage map [19]. Therefore, we considered that some of the SNPs on the *F.* × *ananassa* genomes derived from non-*F. vesca* ancestral species might have been missed in this study, resulting in several LGs of shorter length. The design of an expanded SNP platform including the SNPs missed in this study will be needed to improve the accuracy of the distinction of subgenome-specific regions.

### Characteristics of the ‘Reikou’ S_1_ linkage map

The total length of the ‘Reikou’ S_1_ linkage map was longer than that of previous maps (2050 cM in Bassil et al. [17] and 1820 cM in Sargent et al. [10]). One of the reasons for the longer length of the present map was considered to be the larger number of mapped SNPs compared to the previous studies (there were 6593 SNPs in Bassil et al. [17] and 8407 SNPs in Sargent et al. [10]). In addition, we considered that SNP calling errors may have caused an unpredictable small map interval and an overestimation of the genetic length. SNP calling errors are commonly observed both in SNP array systems and when using a dd-RAD seq approach [29]. Genotype imputation is generally performed when an SNP array is used to decrease the influence of calling error. Although imputation for solitary missing data was performed in this study, it was difficult to correct all the SNP calling errors.

It was expected that each of the four LGs representing correspondence would be constructed in each HG of the strawberry linkage map. Four LGs were successfully generated for five of the seven HGs, whereas more than four LGs were constructed for two HGs (HG1 and HG6), resulting in fragmentation of the LG(s). Of the fragmentized LGs, LG6A-1 and LG6A-2 were combined, because both showed large numbers of Y-N haplotypes (i.e., corresponding to the *F. vesca* genome) and mapped non-overlapped positions on the *F. vesca* genome. No significant evidence for connection was suggested for the other fragmentized LGs. We considered four possible explanations for the smaller number of polymorphic loci on the regions between the fragmentized LGs: (i) the number of designed SNPs was insufficient due to the non-orthologous regions in the *F. vesca* genome, or repetitive sequences in *F. vesca*, (ii) there were highly homozygous regions because of artificial or non-artificial selection during the development of ‘Reikou’, (iii) there were non-subgenome-specific and highly heterozygous regions (ex. ABABABAB genotype) showing non-disomic segregation patterns, and (iv) high repeat sequences were present. The first case would be improved by adding SNPs as described in the above paragraph, but the latter three cases would be more difficult to respond in the ‘Reikou’ S_1_ population because they would involve plant materials. A comparison between the linkage maps developed by different mapping populations will be needed to overcome the problems described in these latter cases.

### Commonality of mapped SNPs across mapping populations

Only 416 of the 22,207 SNPs were commonly mapped among the three mapping populations, ‘Holiday’ × ‘Korona’, ‘Darselect’ × ‘Monterey’, and ‘Reikou’ S_1_. The number of commonly mapped SNPs was much smaller than expected. This result suggested that there was high specificity of SNPs across the strawberry varieties. When a mutation rate of 1.5 × 10^−8^ per site per year is employed [30], the number of point mutations that occur on the strawberry genome is estimated to be 10.6 - 10.8 per a year (the genome size is presumed to be 708–720 Mb [4, 5]). Strawberry breeding was started approximately 150 years ago in Japan, and the breeding materials have been imported from Europe and/or US. The phylogenetic analysis using SSR markers revealed that the clusters for the Japanese cultivars are separated from those of European and/or US cultivars (Isobe et al. unpublished). The large distinction between the materials would be one of the reasons for the smaller number of commonly mapped SNPs. In turn, the small number of commonly mapped SNPs across the three populations would prevent any detailed comparison among the linkage maps. However, because the IStraw90 Axiom® SNP array was commonly used as the genotyping platform in the three populations, the genotypes of the 22,207 SNPs were already obtained in each population. Therefore, comparisons and integration of the three maps would be possible by using haplotypes consisting of the 22,207 SNPs mapped on at least one of the three populations. It would thus be possible to make a comparison by using the haplotypes plotted using the 22,207 SNPs.

## Conclusion

In the present study, we constructed a strawberry linkage map consisting of 31 LGs with a total of 11,574 loci, including 8999 codominant loci. Our results suggest that candidate subgenome-specific loci were randomly located across the genomes. Moreover, comparative mapping between the *F. vesca* genome and the present linkage map revealed small- to large-scale structural variation among the subgenomes. There is a strong demand for strawberry pseudomolecules for the purposes of genetic and genomic analysis in octoploid strawberry, though the *F. vesca* genome sequence has also greatly contributed. We previously reported the first octoploid strawberry genomes assembled with the Illumina and Roche 454 reads [6], although we are not yet satisfied with their quality. The present linkage map is expected to contribute to the construction of pseudomolecules as well as to their utilization in molecular genetics and breeding.

## Additional files

Additional file 1: List of the tested SSR markers. (XLSX 81 kb)

Additional file 2: Expected segregation patterns of polymorphic loci in the S_1_ and F_1_ mapping populations in allo-octoploid species. A and B indicate bi-alleles while O indicates a null allele on the given locus. (XLSX 38 kb)

Additional file 3: Positions of SNP and SSR loci in the ‘Reikou’ S_1_ population, and probe and primer sequences of SNPs and SSRs, respectively. (XLSX 1445 kb)

Additional file 4: Detailed summary statistics of the ‘Reikou’ linkage map. (XLSX 53 kb)

Additional file 5: Graphical view of syntenic relationship between the ‘Reikou’ linkage map and *F. vesca* genome (v2.0.a1) or *F. iinumae* linkage map [25]. Outer pink, green and yellow arks show the LGs of the ‘Reikou’ linkage map, the chromosomes of *F. vesca*, and the LGs of *F. iinumae*, respectively. Syntenic loci between the two species are connected by colored lines*.* Scales represent the genetic position on LGs (cM) or physical position on chromosomes (Mb). (TIFF 4364 kb)

Additional file 6: Comparison between the physical positions on *F. vesca* (v2.0.a1) pseudomolecules and the ‘Reikou’ linkage map of the SNP and SSR loci. PHR-SNP, NMH-SNP, and SSR markers are shown with blue, red, and green dots, respectively. Data for the linkage groups LG6A-1 and LG6A-2 are joined with an artificial gap of 10 cM. (TIFF 5454 kb)

Additional file 7: SNP positions and numbers of the mapped Illumina-reads on the ‘Reikou’ linkage map. Black, blue, and red bars represent the positions of all SNPs, PHR-SNPs, and NMH-SNPs, respectively. Blue and red dots show the number of mapped Illumina reads on the probe sequences of PHR- and NMH SNPs, respectively. Probability values by Student’s *t*-test (**, *p* < 0.01; *, *p* < 0.05) for the comparison between the reads on the flanking sequences of PHR- and NMH-SNPs are indicated in the lower right of the boxes. LG6A-1 and LG6A-2 are combined with an artificial 10 cM gap. (TIFF 7257 kb)

Additional file 8: The ratio of the probes for the mapped Illumina read depth onto the probe sequences of PHR- and NMH-SNPs. Ratio in PHR- and NMH-SNPs are indicated in the blue and the red lines, respectively. (TIFF 1674 kb)

Additional file 9: Number of haploSNPs on the linkage groups of the ‘Reikou’ map. The green, gray, yellow, and blue bars indicate the number of haploSNP types categorized as Y-N (*F. vesca* matched but not *F. iinumae*), Y-Y (both *F. vesca* and *F. iinumae* matched), N-Y (*F. vesca* not matched but *F. iinumae* matched), and N-N (neither *F. vesca* nor *F. iinumae* matched), respectively. Numbers of haploSNPs were counted separately for fragmentized LGs of HG6, LG6A-1 and LG6A-2. (TIFF 2389 kb)

Additional file 10: Number of commonly mapped SNPs among the ‘Reikou’ linkage map and the two previously published linkage maps constructed with the Axiom® array. (TIFF 2866 kb)

## Abbreviations

AFLP: Amplified fragment length polymorphism
BLAST: Basic local alignment search tool
CRBT: Call rate below threshold
DArT: Diversity array technology
ddRAD seq: Digest restriction-associated DNA sequencing
DM: ‘Darselect’ × ‘Monterey’
HG: Homoeologous group
HK: ‘Holiday’ × ‘Korona’
LG: Linkage group
LOD: Logarithm of odds
MHR: Mono high resolution
NMH: No minor homozygote
OTV: Off Target Variant
PHR: Poly high resolution
QC: Quality control
RAPD: Random amplified polymorphic DNA
SNP: Single nucleotide polymorphism
SSR: Simple sequence repeat
STS: Sequence tagged site
TBE: Tris- borate- ethylenediaminetetraacetic acid
