# Supplementary figures and images for: Discrimination of candidate subgenome-specific loci by linkage map construction with an S1 population of octoploid strawberry (Fragaria × ananassa)
Source: BMC Genomics. 2017 May 12;18:374. doi: 10.1186/s12864-017-3762-y (PMC5429521; doi:10.1186/s12864-017-3762-y)

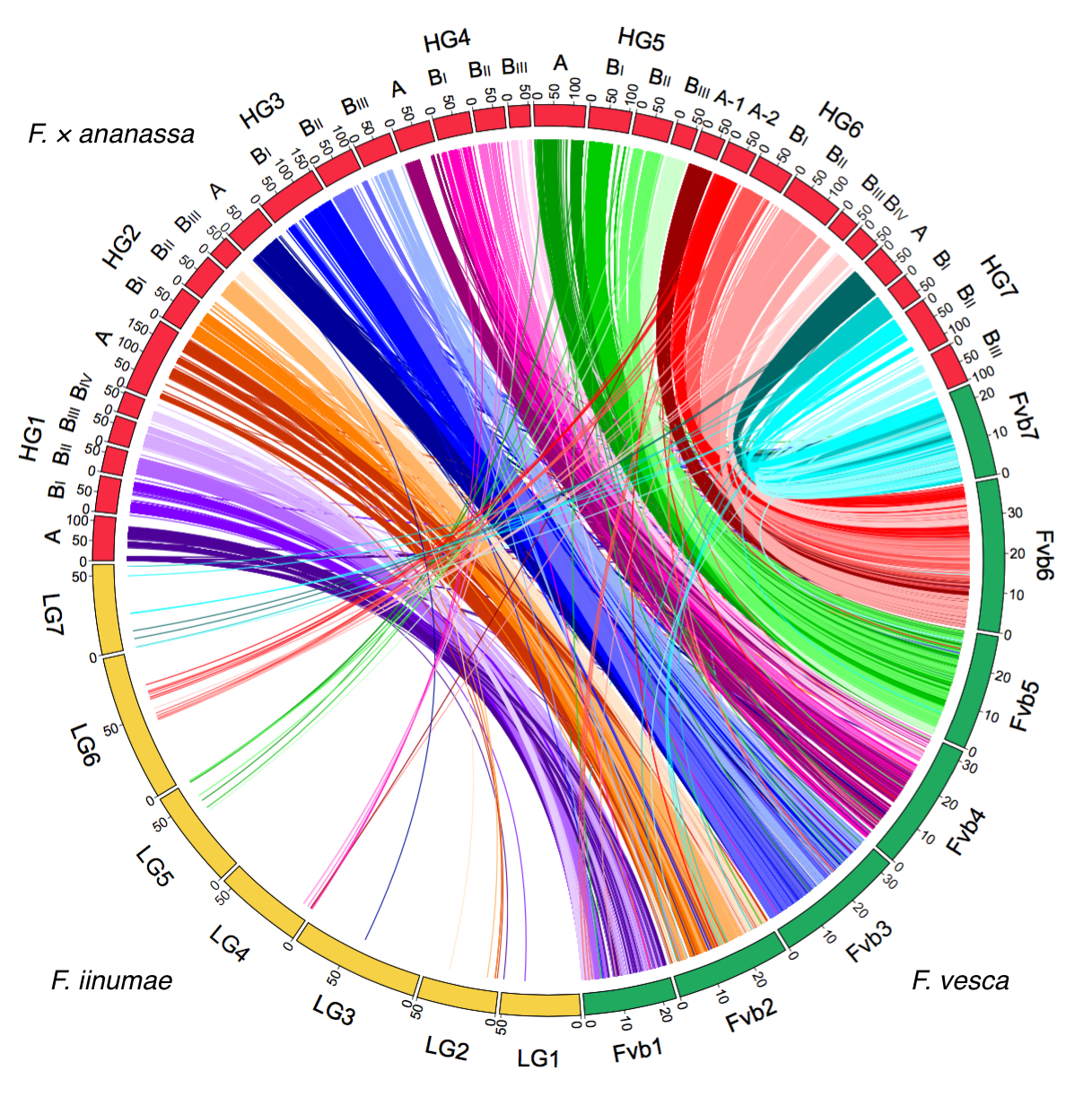

Supplement: Supplementary file 5 — Graphical view of syntenic relationship between the ‘Reikou’ linkage map and F. vesca genome (v2.0.a1) or F. iinumae linkage map [25]. Outer pink, green and yellow arks show the LGs of the ‘Reikou’ linkage map, the chromosomes of F. vesca, and the LGs of F. iinumae, respectively. Syntenic loci between the two species are connected by colored lines. Scales represent the genetic position on LGs (cM) or physical position on chromosomes (Mb). (TIFF 4364 kb) [file 12864_2017_3762_MOESM5_ESM.tiff]

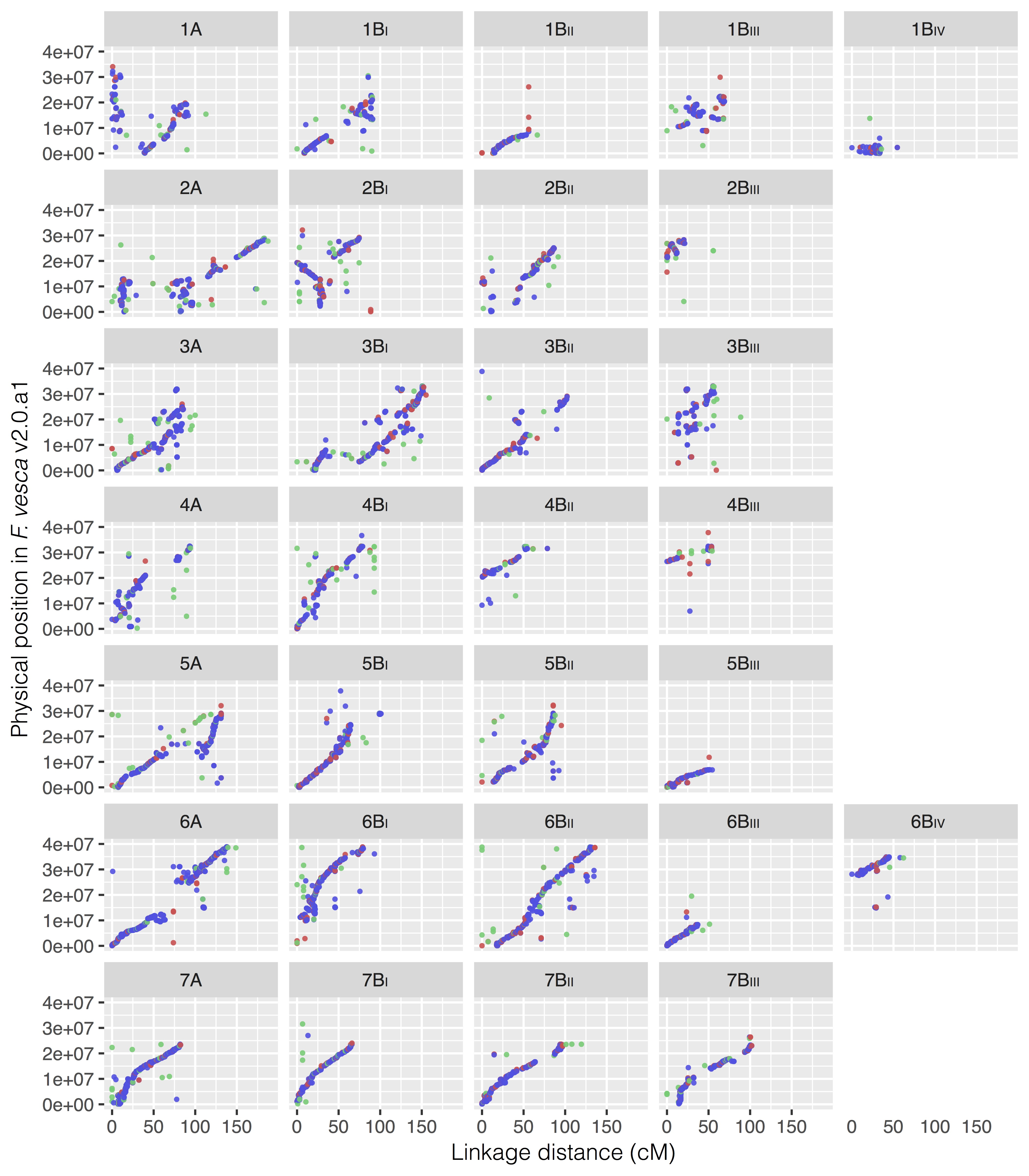

Supplement: Supplementary file 6 — Comparison between the physical positions on F. vesca (v2.0.a1) pseudomolecules and the ‘Reikou’ linkage map of the SNP and SSR loci. PHR-SNP, NMH-SNP, and SSR markers are shown with blue, red, and green dots, respectively. Data for the linkage groups LG6A-1 and LG6A-2 are joined with an artificial gap of 10 cM. (TIFF 5454 kb) [file 12864_2017_3762_MOESM6_ESM.tiff]

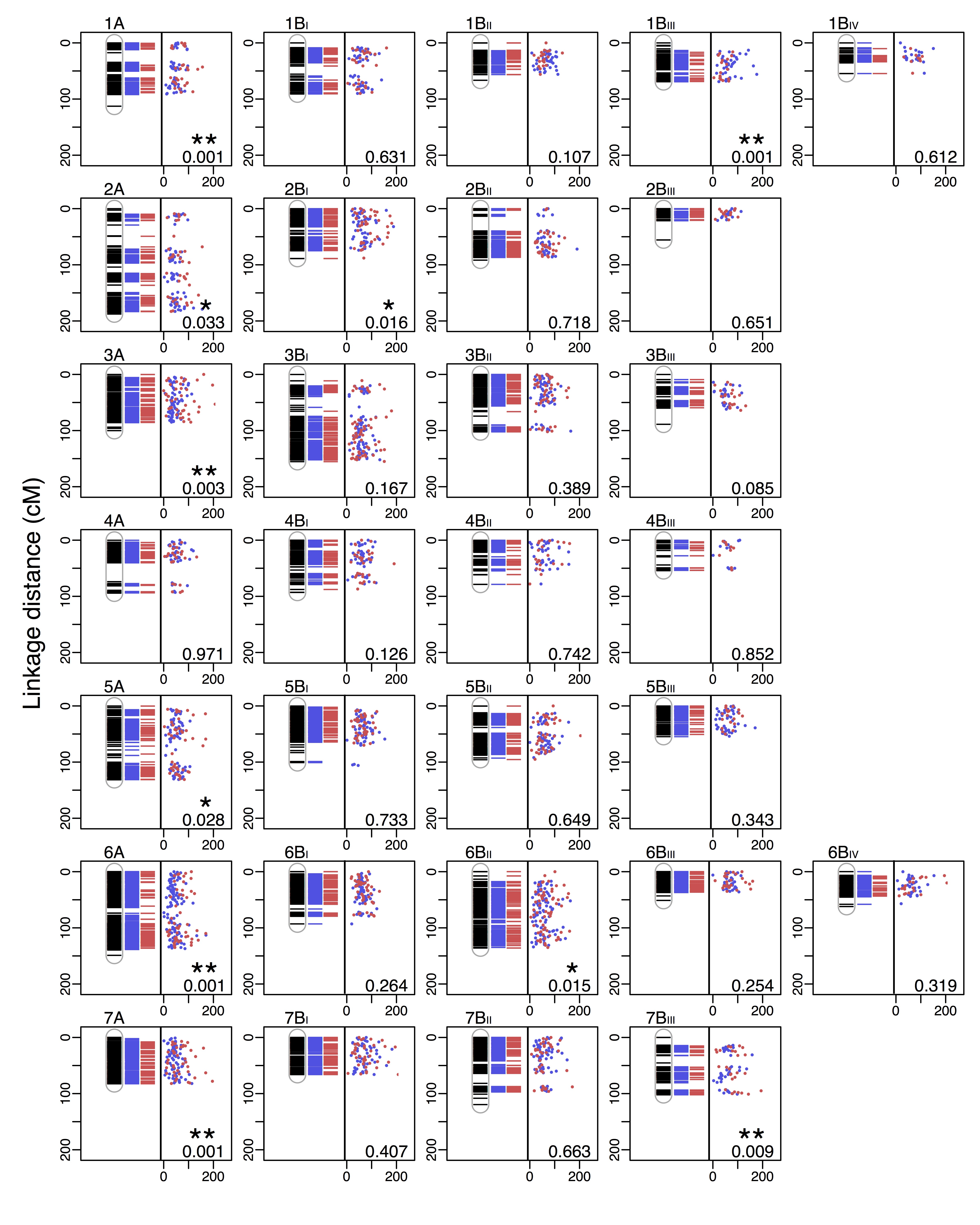

Supplement: Supplementary file 7 — SNP positions and numbers of the mapped Illumina-reads on the ‘Reikou’ linkage map. Black, blue, and red bars represent the positions of all SNPs, PHR-SNPs, and NMH-SNPs, respectively. Blue and red dots show the number of mapped Illumina reads on the probe sequences of PHR- and NMH SNPs, respectively. Probability values by Student’s t-test (**, p < 0.01; *, p < 0.05) for the comparison between the reads on the flanking sequences of PHR- and NMH-SNPs are indicated in the lower right of the boxes. LG6A-1 and LG6A-2 are combined with an artificial 10 cM gap. (TIFF 7257 kb) [file 12864_2017_3762_MOESM7_ESM.tiff]

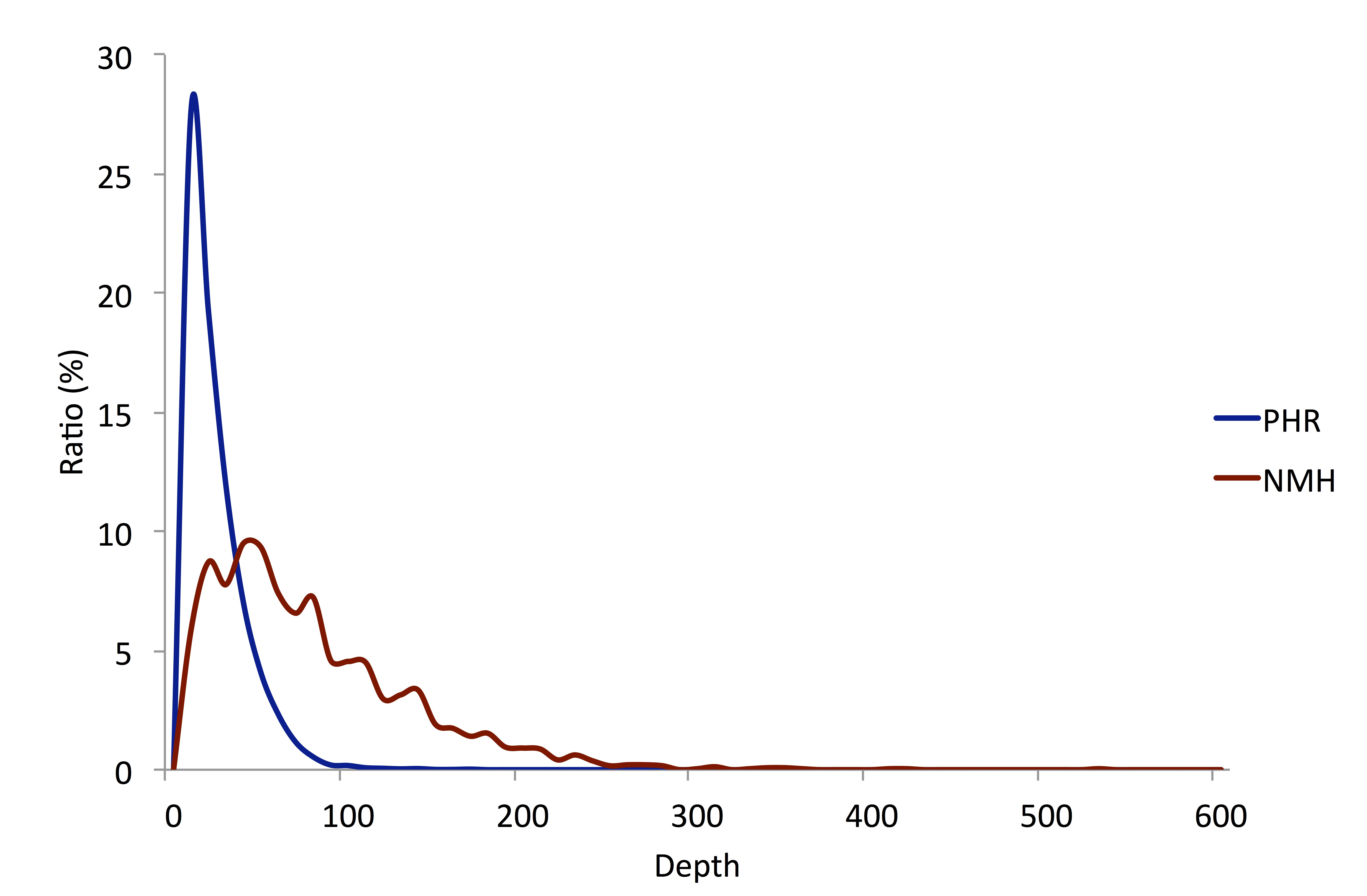

Supplement: Supplementary file 8 — The ratio of the probes for the mapped Illumina read depth onto the probe sequences of PHR- and NMH-SNPs. Ratio in PHR- and NMH-SNPs are indicated in the blue and the red lines, respectively. (TIFF 1674 kb) [file 12864_2017_3762_MOESM8_ESM.tiff]

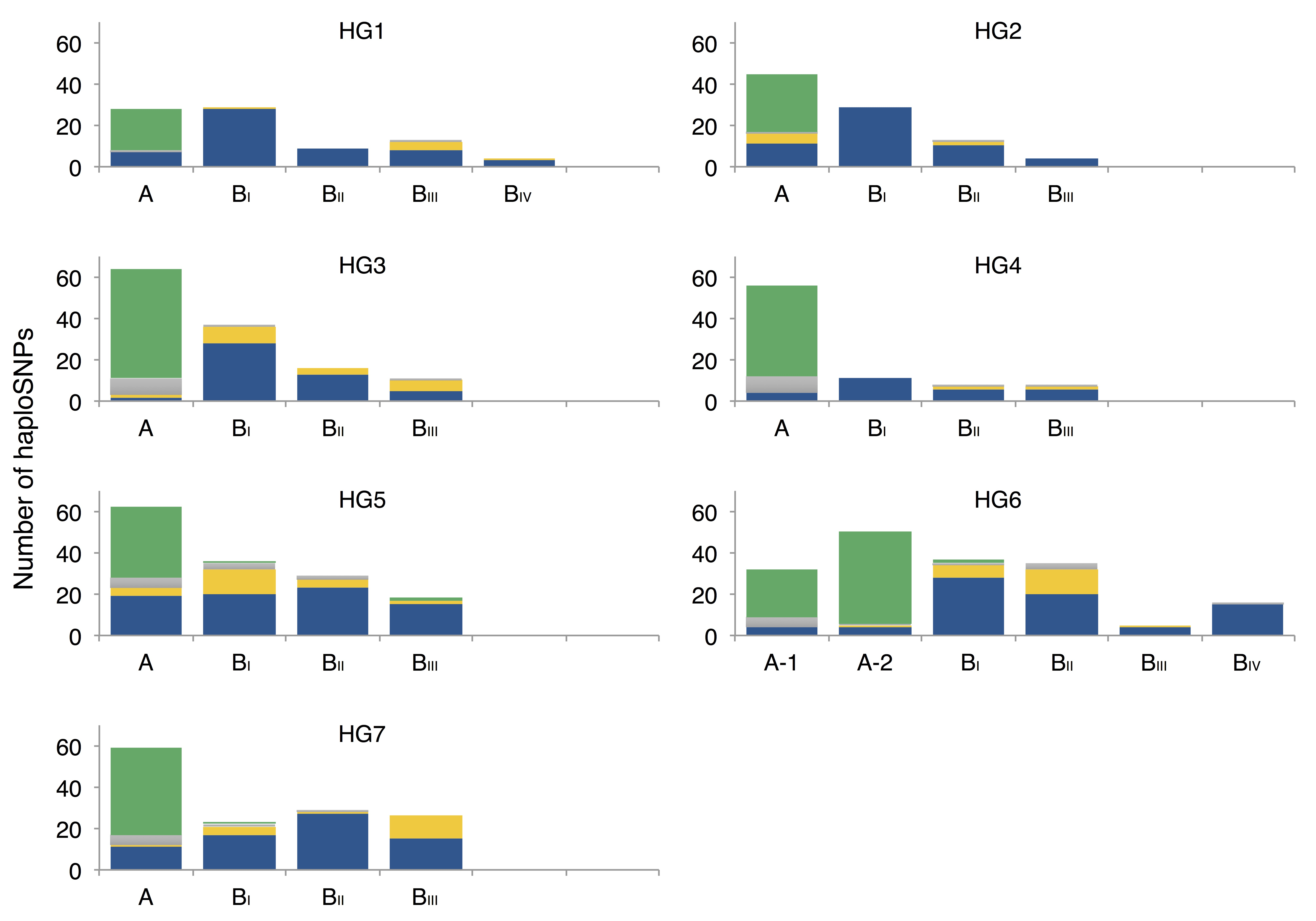

Supplement: Supplementary file 9 — Number of haploSNPs on the linkage groups of the ‘Reikou’ map. The green, gray, yellow, and blue bars indicate the number of haploSNP types categorized as Y-N (F. vesca matched but not F. iinumae), Y-Y (both F. vesca and F. iinumae matched), N-Y (F. vesca not matched but F. iinumae matched), and N-N (neither F. vesca nor F. iinumae matched), respectively. Numbers of haploSNPs were counted separately for fragmentized LGs of HG6, LG6A-1 and LG6A-2. (TIFF 2389 kb) [file 12864_2017_3762_MOESM9_ESM.tiff]

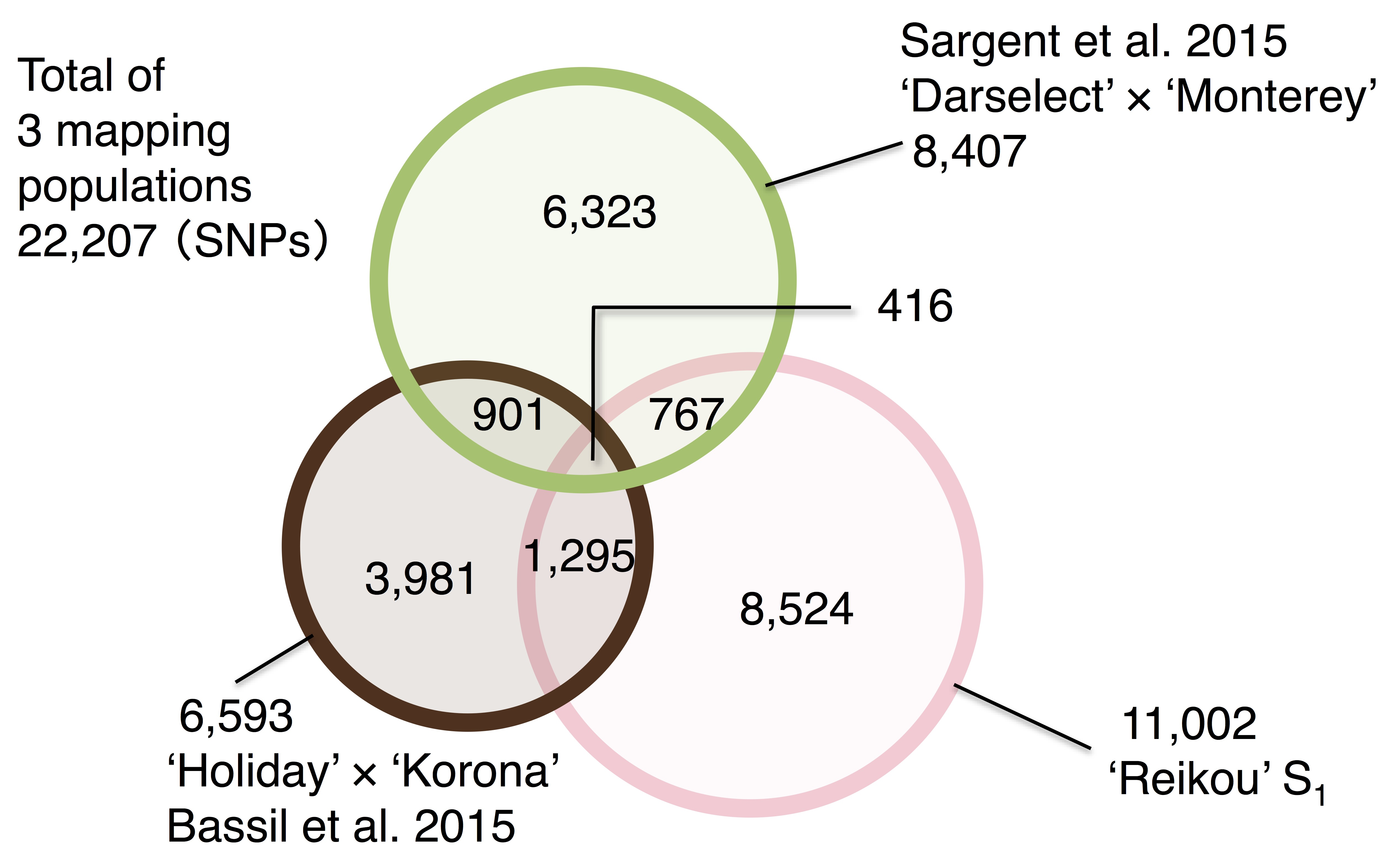

Supplement: Supplementary file 10 — Number of commonly mapped SNPs among the ‘Reikou’ linkage map and the two previously published linkage maps constructed with the Axiom® array. (TIFF 2866 kb) [file 12864_2017_3762_MOESM10_ESM.tiff]
